# Edible Insects: How to Increase the Sustainable Consumption Behavior among Restaurant Consumers

**DOI:** 10.3390/ijerph18126520

**Published:** 2021-06-17

**Authors:** Jinsoo Hwang, Jinkyung-Jenny Kim

**Affiliations:** 1 The College of Hospitality and Tourism Management, Sejong University, 98 Gunja-Dong, Gwanjin-Gu, Seoul 143747, Korea; jhwang@sejong.ac.kr; 2 School of Hotel and Tourism Management, Youngsan University, 142 BansongBeltway, Haeundae-Gu, Busan 48015, Korea

**Keywords:** an edible insect restaurant, theory of planned behavior, product knowledge, behavioral intentions

## Abstract

Edible insects deserve increased attention as green food source in today’s society and more restaurants embrace them to promote sustainable consumption behavior. This study was design to explore how consumers’ behavioral intentions to use edible insect restaurants were formed based on the theory of planned behavior (TPB) model. Furthermore, the study attempted to deepen TPB by including the moderating role of product knowledge. A total of 440 samples were collected by online survey in South Korea, and the results of structural equation modeling found that all of the hypotheses have been statistically accepted. Additionally, the results of multiple group analysis indicated that product knowledge moderated the link between subjective norm and behavioral intentions. On a basis of the analysis results, we provided significant theoretical implications and practical implications how to increase future sustainable food consumption intention.

## 1. Introduction

The eyes of the world are on the environment and many people are expressing the needs to limit the environmental burden from food production and consumption [1,2]. Livestock farming has resulted in the most pressing environmental problem which includes global climate and soil acidification [3]. Hence, scholars and industry professionals have proposed various mitigation strategies which aim to reduce environmental damage in the livestock sector [4]. On the other hand, the growth of the global population indicates that the conventional sources of protein will soon be insufficient to fulfill increased food demands [5]. That clearly denotes that a new direction is required to foster sustainability and food security simultaneously. With this respect, practitioners have suggested alternative eco-friendly nutrient-dense food sources such as plant-based products which can replace livestock [6]. Among these substitutes, edible insects are noticeable since they are rich in nutrition but with a reduced environmental footprint compared to traditional breeding of beef, pork, or poultry [7,8,9]. Likewise, Aravind [10] asserted that the inclusion of insects in human diets is one of the solutions to the problem of food security and sustainability. Following this, the comprehension of driving forces behind behavioral intentions toward an edible insect restaurant is a necessity for its greater market penetration which eventually contributes to increase the sustainable consumption behavior among restaurant consumers.

In fact, insects have been a food staple for centuries [11]. Even though eating insects is not welcomed in several parts of the world, approximately 2000 kinds of insects are edible and they have been consumed by various ethnic groups [12,13]. Edible insects have been recognized to have a superior nutritional value [7,14] and offer more cost effective opportunities [9]. However, most of all, a green element is the overriding consideration of edible insects in our daily diet. In reference to the various benefits of edible insects and the aforementioned needs of pro-environmental food supply in modern days, more restaurants embrace edible insects as the part of innovations as well as greenness of their products [15]. The extant literature includes studies that deal with edible insects to examine the values as a new food source, consumers’ acceptance, or barriers in the consumption [8,16,17,18]. However, only a handful studies have endeavored to predict consumers’ behavioral intentions from the lens of pro-environmental practice towards restaurants offering dishes with edible insects. Given this, the examination of fundamental factors that entice consumers to visit an edible insect restaurant as a good deed in sustainability aspect will be essential.

The theory of planned behavior (TPB) has been extensively employed to explicate the associations among an individual’s belief, attitude, and behavioral intentions [19]. The TPB encompasses a different set of beliefs which elicit attitude, subjective norm, and perceived behavioral control to predict consumers’ behavioral intentions [20,21]. That is, the theory involves not only volitional factors but also components which are not entirely dependent on individuals’ own volition. Likewise, the TPB has been considered as a more comprehensive framework and its superior predicting power of consumer behavior has been validated through many studies in various sectors [22,23,24]. In addition, numerous scholars endeavored to deepen the TPB to better predict consumer behavior and thereby various moderating variables were incorporated into the theory [25,26,27]. Similarly, attempts to adopt the TPB have recently begun in the context of edible insect foods and/or restaurants [14,28]. Nevertheless, these studies are mainly subject to the consumption of insect-based food products and the efforts to comprehend the formation of individuals’ intentions towards the restaurant are rather rare.

Meanwhile, knowledge level is often used to explain if people are ready or reluctant to try edible insects [17,29,30]. For example, Schösler, De Boer, and Boersema [31] examined consumer behavior towards various meat substitutions and concluded that entomophagy is not well accepted by people because of less knowledge of edible insects. Nonetheless, no study has yet empirically investigated the effect of individuals’ knowledge about premium value of edible insects such as superb nutrient profile and ecological contribution. The above discussions outline the gap in the literature which is insufficient evidence to predict the sustainable consumption intentions towards edible insect restaurants. Therefore, the present study nests its discussion within the TPB, one of the prevailing theories in consumer behavior, in consideration of product knowledge which differs from prior studies. More specifically, this study was designed to address (1) the impact of behavioral, normative, and control beliefs on each respective determinant (i.e., attitude, subjective norm, and perceived behavioral control) of behavioral intentions, (2) the influence of attitude, subjective norm, and perceived behavioral control on behavioral intentions, and (3) the moderating effect of product knowledge within the proposed conceptual model. Efforts in responding to these research questions would contribute to deepen the TPB and advance our practical knowledge about the driving forces for the successful penetration of an edible insect restaurant in the market.

The remainder of this research is structured as following. Section 2 discusses existing studies pertaining to the topic and theoretical background of this research. In addition, each study variable is articulated in consideration of the potential relationships among proposed constructs. Consequently, the conceptual research model and hypotheses are presented. Section 3 describes the method this research conducted to test theoretical framework and hypotheses, and Section 4 elaborates the results of analyses. Section 5 presents the discussion and implications based on the analyses results. Section 6 is then followed by study limitation and suggestions for future research. Last, Section 7 provides the conclusion of the research. 

## 2. Literature Review

### 2.1. An Edible Insect Restaurant

Edible insects are sustainable food sources for increased food demands in the future that appeal to the general public on scientific grounds. Edible insects are highly recognized with their significant economic value, nutritional value, and taste value [1,5,9]. In addition, edible insects require less resource and they boast outstanding conversion rates and exceptional fecundity rates throughout the year [8]. That is, edible insects are cost-effective. Edible insects are wealthy sources of amino acids, fat, minerals, protein, and vitamins [7,32]. To be specific, they contain the greater amount of proteins compared to conventional sources of protein which include dairy products and meats [12,33]. They also deliver more fiber, minerals, and omega-3 than beef [34]. Furthermore, the rich flavor and taste are often illustrated the quality of edible insects [9]. However, most of all, edible insects are eco-friendly products. For instance, edible insects emit a lower level of greenhouse gas and ammonia compared to the traditional livestock [2,18]. Similarly, Halloran et al. [35] discussed how edible insect farming is pro-environmental through comparing its degree of global warming potential from the ones from diverse animal sources. In addition, they make a great contribution to the recycling of livestock waste which aids in conserving and protecting the environment [34]. Hence, scholars and global organizations such as United Nations acknowledged the edible insects as a sustainable food system [2,35]. Concretely, Van Huis and Oonincx [2] observed the role of edible insects from sustainability perspective and they determined that edible insects could mediate a main cause of anthropogenic-induced environmental issues.

Recognizing the above-mentioned benefits, edible insects have come to a greater attention and many entrepreneurs have entered in the restaurant industry in promoting dishes with edible insects [15,29]. Edible insects are regarded as a health food in China and twenty to thirty common species are available at many restaurants in Yunnan province and many other parts of China [36]. Grub Kitchen is one of the UK’s more popular edible insect restaurants and they serve crickets, mealworms and grasshopper burgers with fries [37]. The Insect Experience is a restaurant in South Africa where customers are provided with bug-inspired meals such as mopane polenta fries which is made with flour created with mopane worms [38]. Similarly, some restaurants in South Korea specialize with insect-based food items such as bibimbap, a traditional dish, prepared with edible insects [7]. Apparently, there are innovative and green trendsetters in the restaurant industry all over the world.

### 2.2. Theory of Planned Behavior (TPB)

The TPB is an extended model of the theory of reasoned action (TRA) which denoted attitude and subjective norm as two core determinants of individuals’ behavioral intentions [20]. A number of studies have been conducted to explain the association between psychological variables and environmentally friendly behaviors and/or intentions [15,25,39,40]. Of these endeavors, the general consensus is that individuals’ pro-environmental behaviors/intentions are rooted from their fundamental beliefs [23,26]. According to Fishbein and Ajzen [19], individuals’ behavioral intentions are generated by the considerations of consequences resulting from a certain behavior, opinions of the social environment toward a certain behavior, and facilitators and/or barriers in performing a certain behavior. They are linked to behavioral beliefs, normative beliefs, and control beliefs respectively in the framework of the TPB, and these beliefs are influencing factors of key determinants of consumers’ behavioral intentions which are attitude, subjective norm, and perceived behavioral control [20].

One of the primary goals for many hospitality companies is an increase of individuals’ positive behavioral intentions [24]. The concept of behavioral intentions refers the probability to engage in a certain behavior [41]. The TPB has been proven as a comprehensive model which successfully explains the formation of individuals’ behavioral intentions, and the same findings exist in the context of eco-conscious consumer behavior [22,23,26,42]. Likewise, Shin et al. [43] built a conceptual research model using the TPB and its results confirmed that attitude, subjective norm, and perceived behavioral control are core determinants of individuals’ intentions to select organic menu items, which eventually induce their intentions to visit restaurants specialized with organic menu. Ahmad et al. [44] explored how travelers’ intentions towards environmentally friendly destinations are shaped and their findings confirmed attitude, subjective norm, and perceived behavioral control are formed based on their visiting intentions. Hu, Wu, and Chen [45] endeavored to enhance the comprehension of young Chinese people’s intentions towards a low-carbon travel through an extension of TPB.

#### 2.2.1. Belief and Its Effect

In the study conducted by Eagly and Chaiken [46], attitude was explained as a function of individuals’ behavioral beliefs (BB) which reflect the perceived consequences or outcomes of the behavior and their assessment of the outcome evaluation (OE). That is, people generally evaluate the benefits and costs during their decision-making process whether to conduct a specific behavior or not [47]. Either a favorable or unfavorable attitude depends on their evaluation of consequences [20,26,48]. Likewise, individuals may perceive visiting an edible insect restaurant as socially responsible behavior, reducing environmental damage, and supporting sustainable food consumption system, and such behavioral beliefs and outcome evaluation are likely to exert the positive effect on attitude.

Subjective norm, the second predictor of behavioral intentions, is determined by a person’s normative beliefs (NB) regarding what his/her referents think whether he/she should engage in a certain behavior and his/her motivation to comply (MC) to these opinions [21,49]. Thus, when people think that the dining at an edible insect restaurant is environmentally responsible behavior, they tend to persuade others to visit the restaurant. The last determinant of individuals’ behavioral intention in the model of the TPB is perceived behavioral control which is a function of control beliefs (CB) and perceived power (PP). Control beliefs refer to a person’s perception of the presence and/or absence of resources required to complete a specific behavior, and perceived power illustrates an individual’s evaluation of the importance degree of such resources for the achievement of outcomes [50,51]. Much evidence has supported control beliefs and perceived power underlies perceived behavioral control [23,28] and it is expected that similar associations exist in the area of edible insect restaurants. In summary, these strong links between BBiOEi and attitude, and NBjMCj and subjective norm, and CBkPPk and perceived behavioral control have been empirically validated for individuals’ sustainable consumption behavior [23,26].

#### 2.2.2. Sustainable Attitude and Its Effect

Attitude was defined as “the degree to which a person has a favorable or unfavorable evaluation or appraisal of the behavior in question” [20] (p. 188). That is, attitude accounts for a positive evaluation or negative assessment of a specific behavior in future. The abundant evidence in the existing literature indicates that attitude is the salient predictor of individuals’ behavioral intentions across different settings in the hospitality industry [24,28,45,52]. In the same vein, prior research supported this argument in consumers’ pro-environmental behavior. For instance, Han et al. [23] adopted TPB in a green hotel context and their empirical evidences determined the prominent role of attitude in the development of visit intentions. Paul, Modi, and Patel [53] extended the TPB in order to predict the eco-friendly product purchase intentions of Indian consumers and they validated the significant role of attitude in generating individuals’ buying intentions. Jin, Zhao, and Santibanez-Gonzalez [54] examined why today’s consumers choose eco-labeled products, and their findings observed the significant role of attitude toward purchasing eco-labeled products on their intentional purchasing behavior. Similarly, Qi and Ploeger [44] tested individuals’ green food buying intentions using 300 data collected in China. They reported an essential role of attitude in forming behavioral intentions regardless of consumers’ various cultural backgrounds. From this evidence, it can be inferred that a favorable attitude would elicit the intentions to visit a restaurant which specialized in edible insects.

#### 2.2.3. Subjective Norm and Its Effect

Subjective norm was conceptualized as “the perceived social pressure to perform or not to perform the behavior” [20] (p. 188) and it was proposed as one of the indicators of consumers’ behavioral intentions. That is, individuals perceive ideas and opinions of their significant others such as friends, family, or colleagues and these perceptions affect their decision-making. Subjective norm has been largely suggested as it generates the positive influence in the formation of green behavioral intentions [45,46]. For example, Wang et al. [27] conducted a case study in examining driving forces of travelers’ pro-environmental behavior and their results showed that subjective norm increased behavioral intentions. Bae and Choi [14] built their research framework on a basis of the TPB and their analysis using 392 Korean citizens’ responses and discovered the significant impact of subjective norm on individuals’ behavioral intentions toward edible insects. More specifically, they asserted that if a person’s significant others intake edible insects as regular food ingredients, then the individual will follow such behaviors. Cembalo et al. [55] investigated consumer food choice after an environmental scandal in Italy, and their results showed that consumer intentions are highly predicted by subjective norm. Even though some of scholars determined the insignificant effect of subjective norm on individuals’ intentions (e.g., [22,28]), this study is centered on the ecological aspect of an edible insect restaurant and thus, it posited that subjective norm would positively elicit the intentions towards restaurants featuring pro-environmental products.

#### 2.2.4. Perceived Behavioral Control and Its Effect

According to Ajzen [20] (p. 122), perceived behavioral control refers to “the perceived ease or difficulty of performing the behavior”. Numerous studies tested the role of perceived behavioral control in forming pro-environmental consumer behavior and a critical influence of perceived behavioral control was successfully validated in the domain of hospitality [46,53]. Chen and Hung [22] endeavored to identify the factors influencing individuals’ acceptance of eco-friendly products and they confirmed the critical role of perceived behavioral control in forming behavioral intentions to buy green products. Carfora et al. [25] surveyed people who make household decisions and they demonstrated that perceived behavioral control played a decisive role in generating individuals’ environmentally friendly intentions. Menozzi et al. [28] measured the impact of perceived behavioral control in accepting novel food products which contain edible insects. Their experiments determined that perceived behavioral control is one of the important predictors of individuals’ intentions. Hwang, Kim, et al. [26] employed Ajzen’s TPB framework to comprehend individuals’ intention formation in the foodservice innovation, and their analysis results using 406 samples displayed the strong association between perceived behavioral control and behavioral intentions. Hu et al. [47] studied younger generations’ decision making process in the tourism context, and their findings indicated that the perception of young people about their high ability of control over their behavior exerted a significant influence on their pro-environmental travel choice. Accordingly, it is anticipated that when individuals have confidence and proper resources in visiting restaurants offering foods made by edible insects, there are high possibilities that these individuals will visit an edible insect restaurant. 

### 2.3. Product Knowledge

Product knowledge was described as “the amount of accurate information held in memory as well as self-perceptions of product knowledge” [56] (p. 258). Individuals’ product knowledge level affects the process of purchase behavior since consumers with a high or low degree of product knowledge differ in their evaluation of products/services which in turn influence decision-making [17,24,31,57]. Hence, it can be said that product knowledge is one of the influencing factors and particularly its moderating role has been identified in the association between individuals’ cognitive evaluation and their behavioral intentions. For example, Fu and Elliott [57] investigated the effect of product knowledge in forming consumers’ buying intentions. Their analysis results based on 312 responses revealed that product knowledge moderates the influence of attitude and subjective norm on purchase intentions. Kim and Hwang [24] tested consumers’ ecological intention formation in the field of drone food delivery services and employed product knowledge as an important moderator. Their results successfully confirmed the moderating impact of product knowledge in the link between attitude and behavioral intentions.

According to the report presented by UNFAO, the principle strategy for the success of edible insect consumption is to educate consumers about the environmental and nutritional benefits of edible insects [9,58]. Similarly, Megido et al. [29] examined the Belgium consumers’ adoption level of edible insects in consideration of preliminary knowledge of entomophagy. Their analysis results showed that individuals older than 45 years appreciated insect-based food more than other age groups and it was justified by the better knowledge about edible insects in older people. Sogari et al. [30] explored individuals’ knowledge and attitude in the context of entomophagy in Italy and they adopted a qualitative approach to the group of young foodies who study gastronomy and food science. They specifically tested the knowledge on entomophagy which involves environmental, nutritional, sensory, and social aspects, and their results discovered the group of people with more knowledge exhibited a better acceptance of entomophagy. Liu et al. [17] investigated factors influencing the buying behavior and consumption of edible insects and their analysis of 614 Chinese consumers denoted that knowledge level is a salient factor. Concretely, their results showed a one-level increase in individuals’ knowledge enhances the possibility of buying edible insects by twenty percent. The authors then suggested educational programs to improve individuals’ knowledge in order to increase the chance of purchasing insect-based food. More recently, Wang et al. [59] studied the role of waste management knowledge in the formation of consumers’ pro-environmental behavior and they tested the moderating effect of knowledge in the relationship between attitude and intention. Thus, the strength of the links among constructs rooted from the TPB may differ across high and low product knowledge group.

### 2.4. Proposed Conceptual Model and Research Hypotheses

**Figure 1:**
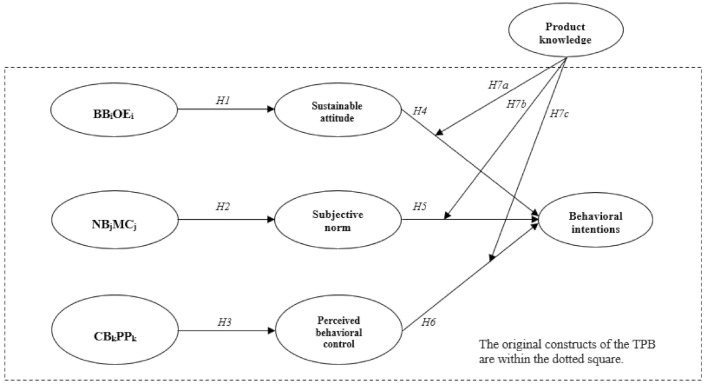
Proposed conceptual model. Note: BB = behavioral beliefs, OE = outcome evaluation, NB = normative beliefs, MC = motivation to comply, CB = control beliefs, and PP = perceived power.

A thorough review of existing studies and evidence, this study propose conceptual framework (Figure 1), and nine hypotheses are formulated. Hypotheses 1–6 postulated the causal relationships among study constructs rooted from the TPB in forming pro-environmental intentions in the context of an edible insect restaurant. In addition, Hypotheses 7a–c are about the impact of proposed moderator, which is product knowledge.

**Hypothesis** **1** **(H1).** *BBiOEi positively promotes sustainable attitude.*

**Hypothesis** **2** **(H2).** *NBjMCj positively promotes subjective norm.*

**Hypothesis** **3** **(H3).** *CBkPPk positively promotes perceived behavioral control.*

**Hypothesis** **4** **(H4).** *Sustainable attitude positively promotes behavioral intentions.*

**Hypothesis** **5** **(H5).** *Subjective norm positively promotes behavioral intentions.*

**Hypothesis** **6** **(H6).** *Perceived behavioral control positively promotes behavioral intentions.*

**Hypothesis** **7a** **(H7a).** *The effect of sustainable attitude on behavioral intentions is moderated by product knowledge.*

**Hypothesis** **7b** **(H7b).** *The effect of subjective norm on behavioral intentions is moderated by product knowledge.*

**Hypothesis** **7c** **(H7c).** *The effect of perceived behavioral control on behavioral intentions is moderated by product knowledge.*

## 3. Methodology

### 3.1. Measurement Items

All of the constructs, such as behavioral beliefs, normative beliefs, control beliefs, outcome evaluation, motivation to comply, perceived power, and behavioral intentions in the TPB model were measured using a seven-point Likert-type scale that ranged from strongly disagree (1) to strongly agree (7), and they were adapted from prior studies [20,21,26]. In addition, as Ajzen [20]’s suggestion, the overall level of each belief construct was determined by multiplying all measurement items of each belief with its corresponding evaluative components (i.e., ΣBBiOEi, ΣNBjMCj, ΣCBkPPk). Lastly, product knowledge was measured with three items cited from [24] (e.g., I feel quite knowledgeable about edible insect restaurants; when compared to other people, I know a lot about edible insect restaurants; and I know a lot about edible insect restaurants). 

### 3.2. Data Collection

The online survey, one of the methods of the convenience sampling technique, was performed using ‘EMBRAIN’ which is known as the best data collection company in South Korea. In addition, the two news articles and one video showed the status of the edible insect market and how it helps protect the environment. First, invitation emails of the survey were sent to 6479 panels, and 450 respondents joined the survey. A total of 10 samples were deleted from the statistical analysis because of visual inspection and multicollinearity problems. Consequently, this study employed 440 samples for the statistical analysis.

## 4. Data Analysis

### 4.1. Descriptive Statistics

Out of the 440 respondents, 50.5% were males (n = 222) while 49.5% were females (n = 218), and their mean age was 38.12 years old. In terms of monthly household income, the largest group reported (28.9%, n = 127) that they had an income between US$1001 and $2000. In addition, the largest category was married people (52.3%, n = 230). Lastly, the majority of the respondents hold bachelor’s degrees (56.4%, n = 248).

### 4.2. Measurement Model

The confirmatory factor analysis (CFA) results indicated that the model adequately fits the data (χ^2^ = 512.882, df = 168, χ^2^/df = 3.106, *p* < 0.001, NFI = 0.954, CFI = 0.968, TLI = 0.960, RMSEA = 0.069). Factor loading was equal to or greater than 0.620 (*p* < 0.001). Table 1 shows the variables and their standardized factor loadings. In addition, the results of the data analysis indicated that the values of Cronbach’s alpha for all of the constructs were greater than 0.70, indicating a high level of reliability [60].

Table 2 reveals that all reliability values for constructs ranged from 0.847 to 0.980, which suggested that all of the measurement items used in this study are reliable [61]. In addition, the values of average variance extracted (AVE) was higher than 0.50, which indicated a high level of convergent validity [62]. Lastly, the results showed that the values of each squared correlation (R2) between a pair of constructs was below the values of AVE for each construct, which suggested a high level of discriminant validity [62].

### 4.3. Structural Model

**Figure 2:**
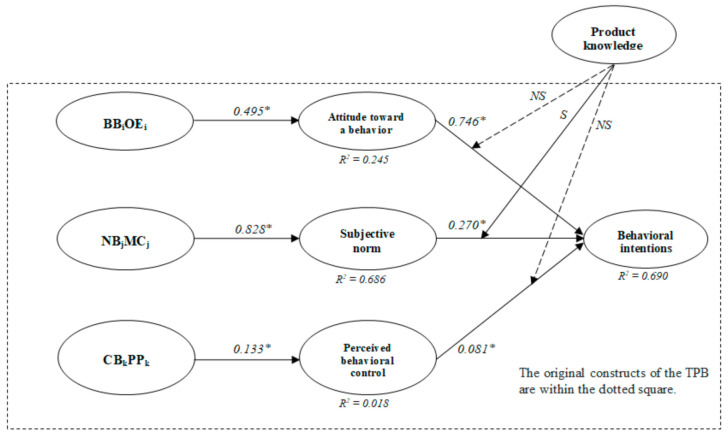
Results of structural equation modeling. Notes 1: BB = behavioral beliefs, OE = outcome evaluation, NB = normative beliefs, MC = motivation to comply, CB = control beliefs, and PP = perceived power. Notes 2: * *p* < 0.05. Notes 3: S = supported and NS = not supported.

The structural equation modeling (SEM) results showed that the proposed model fit the data well (χ^2^ = 827.987, df = 180, χ^2^/df = 4.600, *p* < 0.001, NFI = 0.927, CFI = 0.942, TLI = 0.932, RMSEA = 0.091). Figure 2 shows the standardized, theoretical path coefficients. Table 3 summarizes the results of the hypotheses. The data analysis shows that all six hypotheses were statistically accepted.

### 4.4. Moderating Role of Product Knowledge

In order to check the moderating role of product knowledge, multiple-group analyses were conducted (Table 4). Respondents (n = 440) were separated by two groups based on the median value: a low level of product knowledge group (n = 237) and a high level of product knowledge group (n = 203). The results indicated that the moderating role of product knowledge in the relationship between subjective norm and behavioral intentions was supported (H6a) (Δχ^2^ = 4.476 > Δχ^2^ = 0.5(1) = 3.84, df = 1). More specifically, in terms of the low level of product knowledge group, the path coefficient between subjective norm on behavioral intentions was 0.217 (*p* < 0.05), while the path coefficient was 0.395 (*p* < 0.05) for the high level of product knowledge group. However, H7a and H7c were not significant at the 0.05 level as follows: H7a (Δχ^2^ = 0.343 < Δχ^2^ = 0.05 (1) = 3.84, df = 1) and H7c (Δχ^2^ = 0.564 < Δχ^2^ = 0.05 (1) = 3.84, df = 1). That is, there is no moderating effect of product knowledge in the relationships between (1) sustainable attitude and behavioral intentions and (2) perceived behavioral control and behavioral intentions. 

## 5. Discussions and Implications

### 5.1. Discussions

Of various theories in the consumer behavior, the superior prediction power of the TPB in explaining individuals’ behavior is properly documented in various settings [14,28,53]. The current research adopted the TPB and it successfully validated the applicability of the TPB in comprehending individuals’ intention formation to visit edible insect restaurants. First of all, this study echoed the prior findings [23,28], suggesting the significant effect of belief constructs which are BBiOEi, NBjMCj, and CBkPPk on attitude, subjective norm, and perceived behavioral control. Hence, it can be said that if the focal customers believe dining at edible insect restaurant is pro-environmental behavior which supports sustainable food consumption, and then they are likely to build a positive attitude and a high degree of subjective norm, respectively. Furthermore, when individuals believe the presence of resources to visit an edible insect restaurant and they are confident of using it, they exhibit a high degree of perceived behavioral control.

Second, the results of SEM analysis indicated that attitude, subjective norm, and perceived behavioral control are essential in increasing individuals’ behavioral intentions in the edible insect restaurant context. These findings are in line with prior studies in the sustainability context which confirmed the important roles of these core determinants for the formation of individual intentions [23,26,27]. Specifically, our results echo the findings of Choe et al. [15] which confirmed the significant influence of antecedents stemming from TPB on consumers’ environmentally friendly intentions toward edible insect restaurants. However, there are some studies which illustrated different results from the application of the TPB. For instance, Chen and Hung [22] determined the insignificant association between social impression and individuals’ intentions towards using green products. We infer that our result was different from Chen and Hung’ [22] study since it is subject to a specific product rather than a large collection of different categories of eco-friendly products. Meanwhile, of these three triggers of behavioral intentions, the salient role of attitude was found in this research and it is similar with the studies conducted by Paul et al. [53] and Qi and Ploeger [44].

Last, the assessment to measure the moderating effect uncovered that product knowledge strengthened the casual relationship between subjective norm and behavioral intentions. The moderating role of product knowledge is constantly articulated in consumer behavior, however, it displays somewhat different results [17,24]. For instance, Fu and Elliott [57] confirmed the association between subjective norm and buying intentions weakens for individuals who possess higher product knowledge level. They inferred that individuals are often overconfident about their knowledge and these individuals with a high product knowledge are perhaps reluctant to depend on others’ opinion. In contrast, our findings determined the positive moderating effect of product knowledge in the link between subjective norm and behavioral intentions. That is, a group of people who consider themselves as being relatively knowledgeable are not overconfident in their knowledge level. Nonetheless, a number of other studies confirmed the positive moderating role of product knowledge in the formation of individuals’ ecological intentions. Moreover, the results of this study are supported by studies in the context of edible insects [17,30], which demonstrated the positive role of consumers’ knowledge in their acceptance behavior.

### 5.2. Theoretical Implications

First, this research successfully validated the application of the TPB in an edible insect restaurant context. Even though an edible insect restaurant represents the sustainable food system, this type of restaurant is still regarded as a new concept offering a novel culinary experience, to some extent, in many places [15,39,40]. Understanding the driving forces of consumer behavior is of great importance for the successful penetration of new products/services in the market [23,24]. Despite that, consumers’ intentions towards an edible insect restaurant were seldom explored. In this respect, the current study is one of very few attempts to provide evidence based on the TPB how individuals’ behavioral intentions are formulated from the lens of environmental sustainability in the area of edible insect restaurants. In particular, this study is responding to the limitation of the Choe et al. [15] study which suggested considering additional indicators to improve the predictive power of TPB in examining the formation of consumers’ intentions toward edible insect restaurants. Accordingly, this research contributes to the sustainable development of the restaurant industry, which increases the likelihood of consumers’ pro-environmental behavior.

Second, another uniqueness of this study is an attempt to incorporate product knowledge as an important moderator in the existing conceptual framework of the TPB. The extant studies describe the critical role of product knowledge in forming consumers’ behavioral intentions [17,24]. Likewise, several scholars denoted the significance of individuals’ knowledge in accepting insect-based food [30]. Nonetheless there was no evidence regarding the moderating role of product knowledge in the impacts of volitional and non-volitional factors on behavioral intentions towards an edible insect restaurant. In other words, the present study deepened the TPB in considering a vital moderator and underpinned the essential role of product knowledge in the field of an edible insect restaurant for the first time. Therefore, this research theoretically contributed in explicating the development of consumers’ behavioral intentions towards an edible insect restaurant in great depth.

### 5.3. Managerial Implications

First, the pioneers in the field of an edible insect restaurant should recognize the driving forces behind individuals’ intentions, which are attitude, subjective norm, and perceived behavioral control and establish strategic plans to improve these factors among their target market. Thus, professionals may consider efforts to visualize the consequences of visiting an edible insect restaurant to enhance behavioral beliefs, go viral to increase normative beliefs, and support possible resources to improve control beliefs. Moreover, industry professionals operating an edible insect restaurant are suggested to maximize the marketing opportunities to imprint their ecological value in people’s mind. For instance, the environmental contribution of edible insects can be extensively communicated through various channels to the potential customers to enhance attitude. The partnerships with environmentalist to initiate pubic campaigns would be helpful to strengthen the subjective norm which in turn increases behavioral intentions. Recently, there is a rising celebrity endorsement in the consumptions of edible insect-based foods and it was verified as a powerful tool to induce consumers’ positive behavior [59]. Hence, the cooperation with various influencers would be an effective way to increase individuals’ pro-environmental behavior toward edible insect restaurants. Further, industry practitioners may consider the different type of edible insect restaurants from a fast food chain to the fine dining restaurant, which will offer various pricing points from more locations to aid individuals’ perceived behavioral control.

Second, there are meaningful practical implications that can be extracted from the moderating effect of product knowledge in the link between subjective norm and behavioral intentions. Developing product knowledge is of importance in the domain of an edible insect restaurant and operators in the food industry are suggested to constantly seek for ways to engage with potential customers to educate the substantial values of edible insects [9,59]. Therefore, as part of greener practices in the restaurant industry, running a series of educational forums in the community level would be effective to enhance their knowledge as well as encourage social influence. Practitioners of edible insect restaurants may consider such programs in conjunction with a food tasting or sharing a recipe in order for people to be more familiar with dishes made by edible insects. Such endeavors would be helpful for people to improve the comprehension of the nutrition and taste value of edible insects. These initiatives can be also arranged by and cooperated with public authorities or world organizations that strive to preserve the environment and improve food safety. In addition, as online support has been proven to be important in the context of edible insects [63], innovative activities through online spaces would create more opportunities to increase subjective norm.

## 6. Limitations and Future Research

The following limitations of this study would guide the direction for future research. First, cultural aspects were not considered in this study. In particular, the inclusion of insects was practiced in traditional Korean medicine and it was also accepted as a regular part of human diet in South Korea [12]. Furthermore, and thereby, several scholars addressed the high possibilities of edible insects from farm to a dining table for the Korean market [7]. Negative perceptions toward edible insects exist in communities where it is not familiar and not part of their culture [8,13]. Therefore, more studies would be required in light of other cultural backgrounds and other regions. Additionally, scholars could consider negative psychological variables to discuss barriers to work on the mitigation measures. Last, in spite of the prevailing adoption of TPB in consumer behavior, there are many endeavors in incorporating external factors to better predict consumers’ behavioral intentions [44,46]. Hence studies in the future are suggested to consider additional variables to explain individuals’ intentions in greater detail.

## 7. Conclusions

Today’s consumers are increasingly realizing environmental issues and challenges to ensuring food security [5,23]. Thus, they seek eco-friendly products in their food purchase and consumption behaviors. The existing knowledge of edible insects suggests economic and nutrient values, and more importantly the ecological value of edible insects [9,15]. Likewise, edible insects have been highly recognized as an innovative solution to the problem in sustainable food system and more entrepreneurs in the restaurant industry began to embrace them and brought them to the table. However, there is no sufficient evidence in explaining the core determinants of consumers’ behavioral intentions toward an edible insect restaurant from the perspective of environmental preservation. Accordingly, this current study sought to deepen the TPB by incorporating product knowledge as an important moderator in the formation of consumers’ behavioral intentions toward an edible insect restaurant. The originality of this research shines through this approach which is distinguished from prior studies. Moreover, several meaningful practical implications were derived from findings which suggest effective ways to increase sustainable consumption behavior among restaurant customers. Despite some limitations, this research is among the first attempt to predict individuals’ pro-environmental intentions in the edible insect restaurant context on a basis of psychological theory.

## Figures and Tables

**Table 1:** Confirmatory factor analysis: items and loadings.

Construct and Scale Item	Standardized Loading ^a^
**Behavioral beliefs (BB)** * **Outcome evaluations (OE)** (Cronbach’s alpa = 0.938)	
BB_i_OE_i_1	0.891
BB_i_OE_i_2	0.919
BB_i_OE_i_3	0.935
**Normative beliefs (NB)** * **Motivation to comply (MC)** (Cronbach’s alpa = 0.979)	
NB_j_MC_j_1	0.958
NB_j_MC_j_2	0.982
NB_j_MC_j_3	0.971
**Control beliefs (CB)** * **Perceived power (PP)** (Cronbach’s alpa = 0.835)	
CB_k_PP_k_1	0.620
CB_k_PP_k_2	0.974
CB_k_PP_k_3	0.797
**Attitude (A)** (Cronbach’s alpa = 0.934)	
SA1	0.927
SA2	0.871
SA3	0.930
**Subjective norm (SN)** (Cronbach’s alpa = 0.976)	
SN1	0.948
SN2	0.985
SN3	0.966
**Perceived behavioral control (PBC)** (Cronbach’s alpa = 0.878)	
PBC1	0.831
PBC2	0.962
PBC3	0.727
**Behavioral intentions (BI)** (Cronbach’s alpa = 0.970)	
BI1	0.947
BI2	0.959
BI3	0.965
Goodness-of-fit statistics: χ^2^ = 512.882, df = 168, χ^2^/df = 3.106, *p* < 0.001, NFI = 0.954, CFI = 0.968, TLI = 0.960, RMSEA = 0.069	

Notes 1: Refer to the Appendix A for the following measurement items: behavioral beliefs, outcome evaluations, normative beliefs, motivation to comply, control beliefs, and perceived power. Notes 2: ^a^ all factors loadings are significant at *p* < 0.001. Notes 3: NFI = normed fit index, CFI = comparative fit index, TLI = Tucker–Lewis index, RMSEA = root mean square error of approximation. Notes 4: The asterisk (*) means multiplication.

**Table 2:** Descriptive statistics and associated measures.

	Mean (SD)	AVE	(1)	(2)	(3)	(4)	(5)	(6)	(7)
(1) BB_i_OE_i_	5.06 (0.87)	0.838	0.939 ^a^	0.304 ^b^	0.121	0.480	0.259	0.453	0.467
(2) NB_j_MC_j_	3.12 (1.29)	0.942	0.092 ^c^	0.980	0.114	0.588	0.827	0.099	0.635
(3) CB_k_PP_k_	4.68 (0.88)	0.656	0.015	0.013	0.847	0.085	0.181	0.131	0.105
(4) SA	4.05 (1.49)	0.828	0.230	0.346	0.007	0.935	0.602	0.396	0.850
(5) SN	3.91 (1.28)	0.934	0.067	0.684	0.033	0.362	0.977	0.093	0.629
(6) PBC	4.91 (1.17)	0.715	0.205	0.010	0.017	0.157	0.009	0.881	0.356
(7) BI	3.69 (1.39)	0.916	0.218	0.403	0.011	0.723	0.396	0.127	0.970

Notes 1: BB = behavioral beliefs, OE = outcome evaluations, NB = normative beliefs, MC = motivation to comply, CB = control beliefs, and PP = perceived power, SA = sustainable attitude, SN = subjective norm, perceived behavioral control = PBC, and BI = behavioral intentions. Notes 2: SD = standard deviation and AVE = average variance extracted. Notes 3: ^a^ composite reliabilities are along the diagonal, ^b^ correlations are above the diagonal, and ^c^ squared correlations are below the diagonal.

**Table 3:** Standardized parameter estimates for structural model.

			Standardized Estimate	*t*-Value	Hypothesis
H1 BB_i_OE_i_	→	SA	0.495	10.463 *	Supported
H2 NB_j_MC_j_	→	SN	0.828	28.300 *	Supported
H3 CB_k_PP_k_	→	PBC	0.133	2.653 *	Supported
H4 SA	→	BI	0.746	20.066 *	Supported
H5 SN	→	BI	0.270	8.912 *	Supported
H6 PBC	→	BI	0.081	2.642 *	Supported
Goodness-of-fit statistics: χ^2^ = 827.987, df = 180, χ^2^/df = 4.600, *p* < 0.001, NFI = 0.927, CFI = 0.942, TLI = 0.932, RMSEA = 0.091					

Notes 1: BB = behavioral beliefs, OE = outcome evaluations, NB = normative beliefs, MC = motivation to comply, CB = control beliefs, and PP = perceived power, SA = sustainable attitude, SN = subjective norm, perceived behavioral control = PBC, and BI = behavioral intentions. Notes 2: * *p* < 0.05. Notes 3: NFI = normed fit index, CFI = comparative fit index, TLI = Tucker–Lewis index, and RMSEA = root mean square error of approximation.

**Table 4:** Results for the moderating role of product knowledge.

		The Low Group		The High Group		Unconstrained Model	Constrained Model	Δχ^2^ (1) = 3.84	Result
		β (*R*^2^)	*t*-Value	β (*R*^2^)	*t*-Value				
H7a	A—BI	0.723 (59.2%)	13.347 *	0.748 (80.5%)	14.783 *	χ^2^ (360) = 1097.042	χ^2^ (361) = 1097.385	Δχ^2^ (1) > 0.343	Not supported
H7b	SN—BI	0.217 (59.3%)	4.781 *	0.395 (81.0%)	10.367 *		χ^2^ (361) = 1101.518	Δχ^2^ (1) < 4.476	Supported
H7c	PBC—BI	0.075 (60.3%)	1.658	0.082 (79.9%)	2.105 *		χ^2^ (361) = 1097.060	Δχ^2^ (1) > 0.564	Not supported

Notes 1: SA = sustainable attitude, SN = subjective norm, PBC = perceived behavioral control, and BI = behavioral intentions. Notes 2: * *p* < 0.05.

## Data Availability

The data will be made available on request.

## Appendix A

1. Behavioral beliefs (using an edible insect restaurant would enable me to…)

Protect our environment.

Be more socially responsible.

Perform environmental friendly practices.

2. Outcome evaluations

Protecting our environment is…

Being more socially responsible is…

Performing environmental friendly practices is…

3. Normative beliefs

My family (or relatives) thinks I should use an edible insect restaurant.

My friends think I should use an edible insect restaurant.

My colleagues (or co-workers) think I should use an edible insect restaurant.

4. Motivation to comply

Generally speaking, how much do you want to do what your family (or relatives) thinks you should use an edible insect restaurant?

Generally speaking, how much do you want to do what your friends think you should use an edible insect restaurant?

Generally speaking, how much do you want to do what your colleagues (or co-workers) think you should use an edible insect restaurant?

5. Control beliefs (Using an edible insect restaurant is more likely to…)

Be expensive.

Take time and effort.

Be inconvenient.

6. Perceived power

The cost of using an edible insect restaurant would influence my decision.

The time and effort to use an edible insect restaurant would influence my decision.

The inconvenience of using an edible insect restaurant would influence my decision.

7. Attitude toward using an edible insect restaurant

Unfavorable—Favorable

Bad—Good

Negative—Positive

8. Subjective norm

Most people who are important to me think I should use an edible insect restaurant.

Most people who are important to me would want me to use an edible insect restaurant.

People whose opinions I value would prefer that I use an edible insect restaurant.

9. Perceived behavioral control

Whether or not I use an edible insect restaurant is completely up to me.

I am confident that if I want, I can use an edible insect restaurant.

I have resources, time, and opportunities to use an edible insect restaurant.

10. Behavioral intentions

I will dine out at an edible insect restaurant.

I am willing to dine out at an edible insect restaurant.

I am likely to dine out at an edible insect restaurant.
